# Multidisciplinary management of tracheal paraganglioma with angiographic embolization and surgical excision: A case report and review of literature

**DOI:** 10.1097/MD.0000000000042712

**Published:** 2025-08-01

**Authors:** Javad Jalili, AhmadReza Amiri, Seyed Ziyaeddin Rasi Hashemi, Sahar Rezaei, Mohammadreza Mikaeeli, Mahsa Karbasi, Sarah Vaseghi

**Affiliations:** a Department of Interventional Radiology, Tabriz University of Medical Sciences, Tabriz, Iran; b Department of Radiology, Tabriz University of Medical Sciences, Tabriz, Iran; c Department of Cardiovascular Surgery, Tabriz University of Medical Sciences, Tabriz, Iran.

**Keywords:** ablation, angiographic embolization, neuroendocrine tumors, tracheal mass, tracheal paraganglioma

## Abstract

**Rationale:**

Tracheal paragangliomas (PGs) are exceedingly rare tumors originating from chromaffin cells. They often present with nonspecific symptoms like dyspnea and hemoptysis, which can mimic more common respiratory conditions such as asthma.

**Patient concerns:**

We present the case of a 58-year-old female initially diagnosed with asthma who later experienced recurrent hemoptysis and worsening dyspnea.

**Diagnoses:**

Rigid bronchoscopy revealed a vegetative mass located 2 cm below the vocal cords, and a biopsy confirmed PG. Cross-sectional imaging identified a lobulated lesion on the posterior tracheal wall.

**Interventions:**

To prevent excessive bleeding, angiographic embolization was performed before bronchoscopic surgical resection, involving super-selective catheterization of the tumor’s arterial feeder.

**Outcomes:**

The embolization successfully reduced vascularity, facilitating safe tumor resection. The patient remains symptom-free at the 1-year follow-up, indicating effective management of the condition.

**Lessons:**

This case highlights the importance of a multidisciplinary approach in diagnosing and treating tracheal PGs. Preoperative embolization is crucial for managing bleeding risks associated with these highly vascular tumors. Detailed insights into the angiographic procedure are provided, along with a systematic review of similar cases.

## 1. Introduction

Tracheal masses, a rare entity in general, are mostly secondary to invasions from other malignant sources.^[1]^ Paragangliomas (PGs), also known as chemodectomas, are rare, mostly benign, primary masses that arise from the chromaffin cells originating from the neural crest. They can therefore be scattered throughout the body. In the head and neck region, they mostly originate from the carotid body, jugulo-tympanic region, vagus nerve, middle ear, orbit, mediastinum, aortic arch, and nose, with the trachea being one of the rarest of those locations.^[2,3]^ In the larynx, PGs can be scattered anywhere along the natural distribution of the superior or inferior paraganglion. Depending on their relative location, they can be classified as supraglottic, glottic, or subglottic. The latter, which are more scarce, originate from the inferior laryngeal paraganglion and can be seen as purely intraluminal (also known as tracheal PGs), extraluminal (thyroid PGs), or both.^[4]^ Histologically, tracheal PGs are similar to pheochromocytomas. However, they produce minimal secretions, making them important not because of the paraneoplastic effects they elicit but because they push or displace other vital organs, a phenomenon known as mass effect.^[5]^ PGs are characterized by hemoptysis, dyspnea, and cough as a result of the aforementioned airway compromise. These clinical presentations render the diagnosis and treatment of patients more challenging because a wide range of diseases share these signs and symptoms.^[1]^ Magnetic resonance imaging (MRI) can demonstrate hypervascularity of PGs, and angiographic studies such as magnetic resonance angiography or computed tomography (CT) angiography are performed to evaluate the vascular supply. Whole-body imaging with CT or MRI can determine the presence of possible synchronous masses.^[6]^ Immunohistochemical tests are also conducted to examine the mutations related to the presenting mass.^[7]^ Surgical resection is the basis of treatment; however, special considerations must be taken due to the hypervascular nature of the tracheal PGs, which makes them sensitive to bleeding even during tissue sampling.^[8]^

In the current literature, the case of a 58-year-old female presenting with asthmatic symptoms with a final diagnosis of tracheal PG is presented. She underwent angiographic embolization before tumor excision. An experienced interventional radiologist performed the angiographic procedure, which is explained in detail. This paper also provides a systematic literature review considering the presenting symptoms, demographic data, and treatment options of tracheal PGs.

## 2. Case report

**Figure 1.**
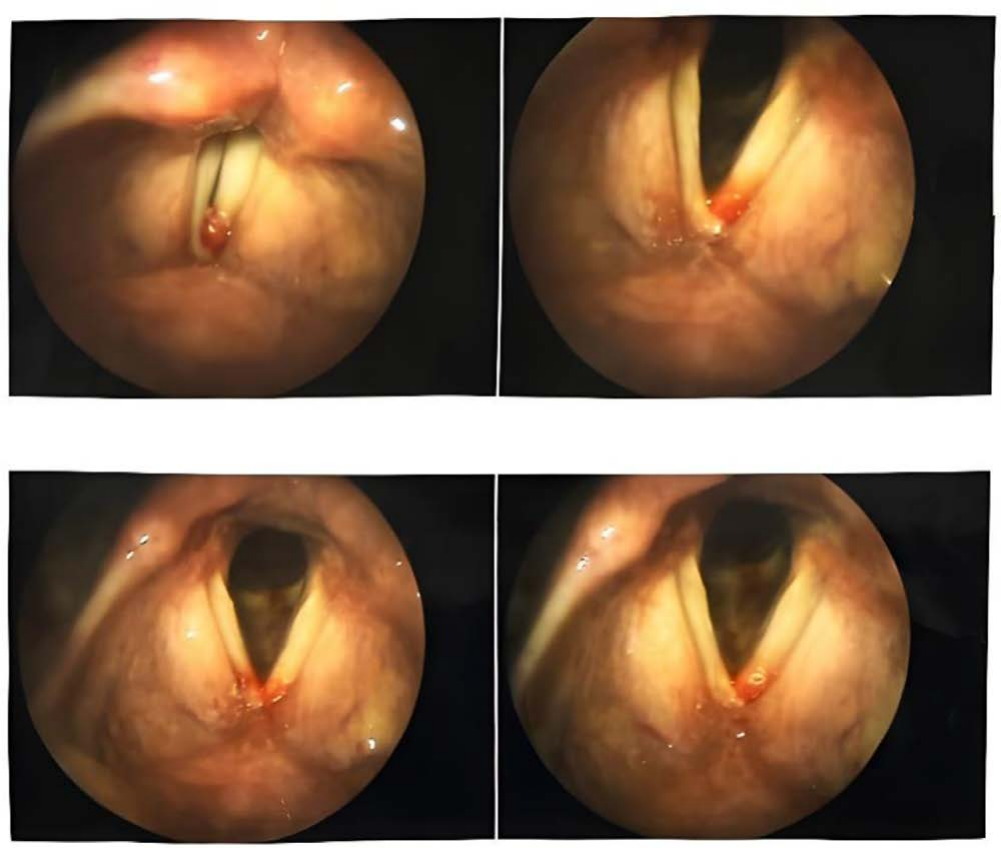
Flexible laryngoscopy exhibiting hemoptysis and mucosal hypertrophy.

A 58-year-old female patient with no history of medical illness other than hypertension presented to our department with dyspnea and wheezing starting 2 years ago. She was under treatment with losartan/hydrochlorothiazide and amitriptyline, and her blood pressure was well controlled. Dyspnea was more prominent at rest and while lying supine. She also experienced a globus sensation. The patient was treated with bronchodilators with a new diagnosis of asthma that failed to improve her overall condition. During the 6 months leading up to her admission, she experienced recurrent episodes of hemoptysis with minute volumes and progressive cough. The patient had no other complaints such as hoarseness or dysphagia. She also reported a weight loss of about 5 kg over the same period. Spirometry studies revealed an obstructive pattern. Three weeks before admission, she experienced massive hemoptysis, cyanosis, and fainting. Laryngoscopy revealed hypertrophic interarytenoid mucosa, hyperactivity of the false vocal cords during phonation, and laryngopharyngeal reflux (Fig. 1). Suspecting a tracheal lesion, a rigid bronchoscopy was performed, and the visualized mass was biopsied. The biopsy sample showed the proliferation of cell nests with sustentacular cells around them and hypervascular stroma, which was suggestive of PG.

**Figure 2.**
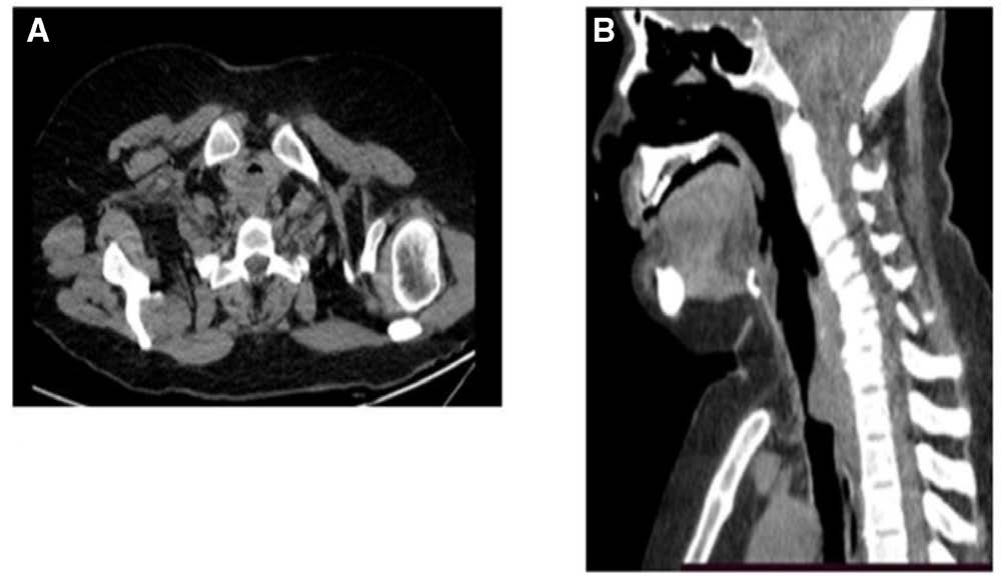
Axial (A) and sagittal (B) non-contrast CT scan images in the mediastinal window showing soft tissue mass narrowing in the tracheal lumen. CT = computed tomography.

**Figure 3.**
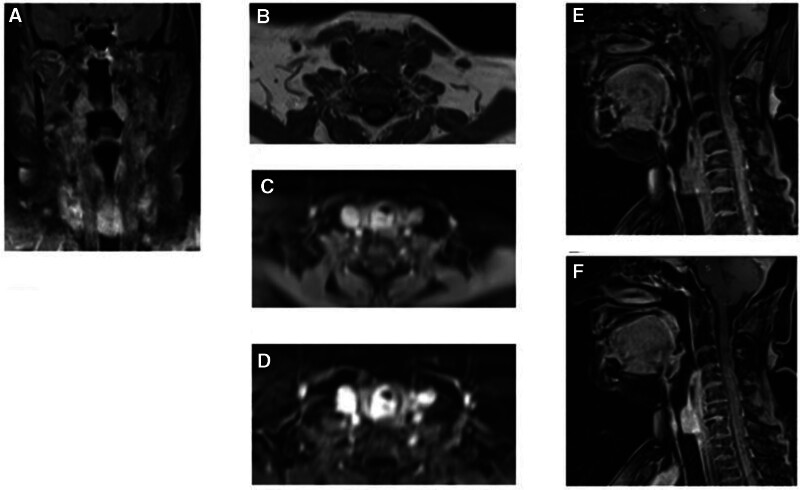
MRI of neck and thorax; coronal T2 (A), axial T1 (B), axial T1 fat sat with contrast (C), subtraction (D), sagittal T1 fat sat (E), and sagittal T1 fat sat with contrast (F) demonstrating a hypervascular mass on the posterior tracheal wall with luminal narrowing. The mass shows iso-intensity on T1W, hyper-intensity on T2W, and marked enhancement on contrast-enhanced images. MRI = magnetic resonance imaging.

Meanwhile, cross-sectional studies were performed to image the lungs and airways. On CT scans, tracheal luminal narrowing with a vanished fat plane between the lesion and the upper esophagus was noted (Fig. 2). The cervical soft tissue and thoracic MRI showed a lobulated and clearly defined lesion of 23 × 20 × 15 mm located at the posterior wall of the subglottic area and the underlying posterior wall of the trachea. It was iso-signal on T_1_ images and high signal on T_2_-weighted images and showed marked homogeneous enhancement after contrast media administration (Fig. 3).

**Figure 4.**
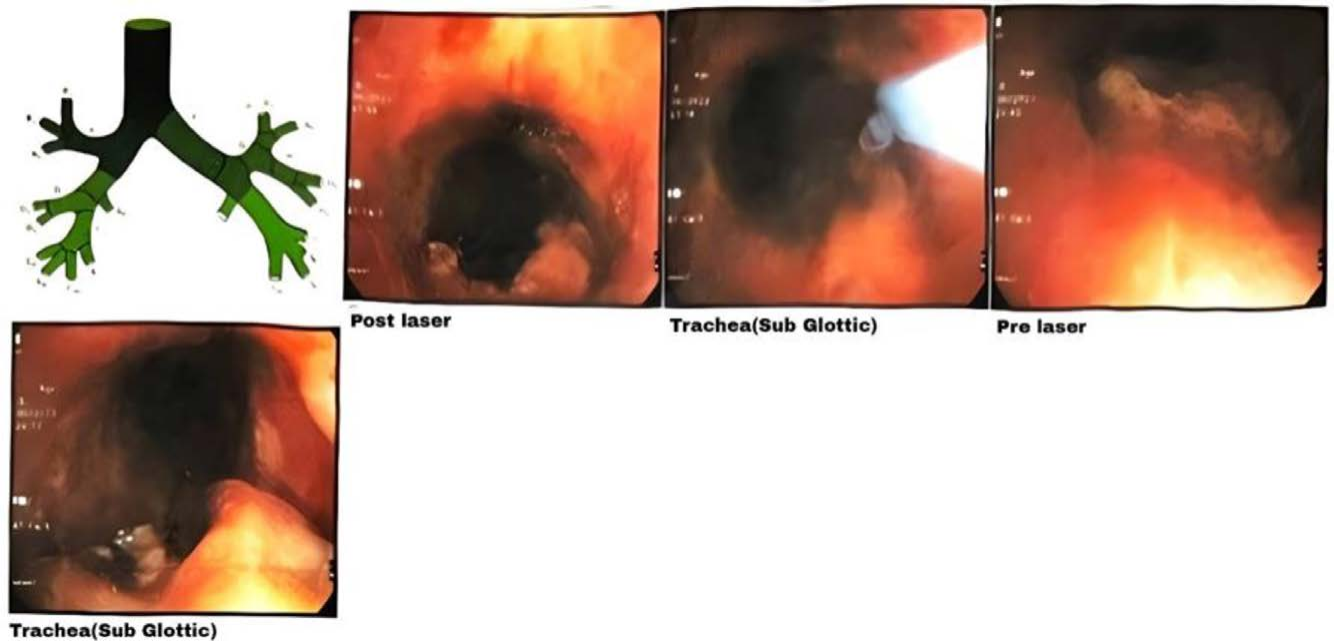
Rigid bronchoscopy images showing subglottic stenosis pre-laser and post-laser ablation.

The patient underwent bronchoscopy multiple times for various purposes other than tissue biopsy. It was conducted to assess the rate of stenosis and to perform repair and reconstruction of the subglottic region using argon plasma coagulation with polyvinyl alcohol (PVA) hydrogel, a biocompatible synthetic polymer commonly used in hemostatic and wound healing applications. The patient underwent 2 separate instances of rigid bronchoscopy in April and May 2023, respectively. The first time, a hypervascular mass approximately 2 cm below the vocal cords with 80% to 90% stenosis was visualized. Argon plasma coagulation was applied to the tumor bed to minimize bleeding and to ablate, managing to decrease the stenosis to 60%. For the second time, the same procedure managed to reduce the stenosis to 40% (Fig. 4). However, the patient continued to experience symptoms.

Given the high vascularity of the tracheal mass and its susceptibility to bleeding, the medical team decided to perform angiographic embolization before tumor excision. The treatment options and associated risks were explained to the patient, and written consent was obtained before the procedure. To ensure optimal aeration of the lungs and to prevent airway compromise due to post-embolization edema, a tracheostomy was performed, and a 7.5 mm tracheostomy tube was fitted in the operating room before performing angiography.

**Figure 5.**
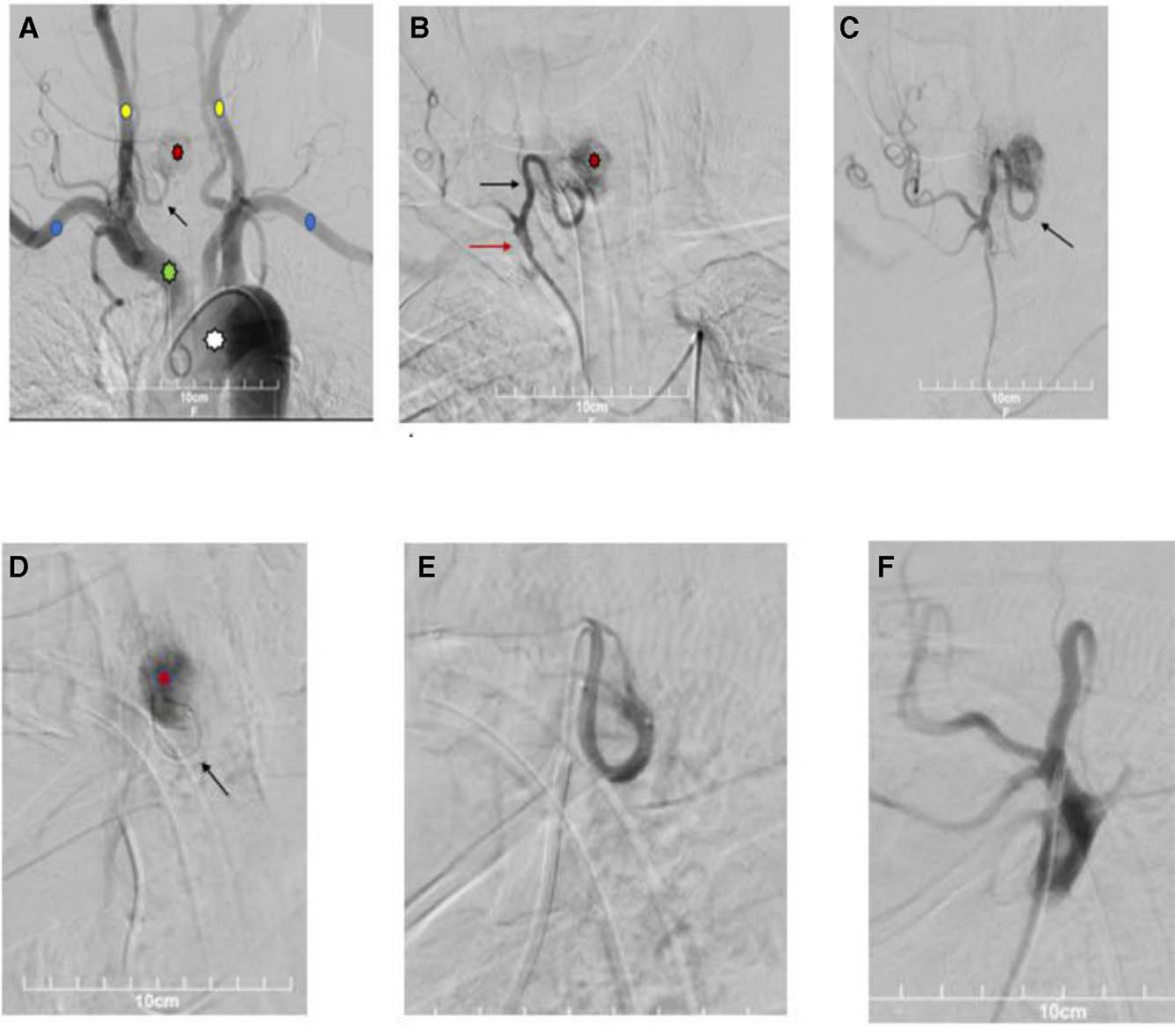
Angiographic studies: (A) Pre-embolization aortography demonstrating the aortic arch (white star), and its main branches, including brachiocephalic (green star), common carotid (yellow dots), and subclavian arteries (blue dots). There is a tumoral blush (red star) close to the midline, which is fed by the right inferior thyroid artery (ITA) (black arrow). (B) The thyrocervical trunk (red arrow), ITA (black arrow), and the enhancing mass (red star) are demonstrated. (C) Selective microcatheterization of the ITA (black arrow) was performed. (D) The super-selective angiography with microcatheter (black arrow) in place (red star). (E and F) Post-embolization angiograms using PVA (particle size of 355–500 μM) demonstrating successful devascularization and elimination of the tumoral blush. PVA = polyvinyl alcohol.

After puncturing the common femoral artery using ultrasound guidance (with Esaote MyLab 40, Esaote S.p.A, Genoa, Liguria, Italy), a 6Fr (Teleflex Incorporated, Arrow International Inc., Reading) percutaneous introducer sheath was utilized to gain access to the femoral artery. Next, using a 6Fr JR angiographic catheter (BIOTEQ, Taipei City, Taiwan) and an AqWire standard hydrophile guidewire (Medtronic, Dublin, Ireland), aortography and selective angiography (using Siemens Artis zee PURE biplane, Siemens Healthineers AG, Erlangen, Bavaria, Germany) of the right common carotid artery, right subclavian artery, right external carotid artery, right vertebral artery, and the right thyrocervical trunk arteries were performed. This revealed a tumor blush anterior to the cervicothoracic junction of the vertebrae fed by the right inferior thyroid artery. After visualizing the tumoral blush and placement of a 6Fr chaperon guiding catheter (Microvention, Aliso Viejo), super-selective catheterization of the tumor’s arterial feeder was achieved using a Headway 27 microcatheter (Microvention) and a Traxcess 14 micro-guidewire (Microvention). Finally, embolization was done using Merit Medical Systems PVA solution (Bearing nsPVA with a particle size of 355–500 μM) to prevent excessive bleeding after the surgical removal of the tumor (Fig. 5). PVA was preferred over other embolic types for devascularization, considering its small size. Smaller-sized particles were not chosen as they may increase the risk of necrosis in neighboring tissues. The tumor was removed using the tip and forceps of a rigid bronchoscope, a technique known as “core out.” Our team’s thoracic surgeon reported that the tumor did not bleed during the surgery and was completely devascularized. No immediate complications, such as hemorrhage, hemothorax, or pneumothorax, were encountered. The patient is currently disease-free after about 1 year.

## 3. Discussion

PGs are slow-growing tumors deriving from extra-adrenal chromaffin cells. Unlike pheochromocytomas, which produce catecholamines, PGs are biochemically silent. Their pattern of distribution in the body is along the paravertebral sympathetic ganglia (thorax, abdomen, and pelvis) and parasympathetic ganglia along the glossopharyngeal and vagal nerve path.^[9]^ PGs are rare tumors (1:30,000–1:500,000 prevalence), and the trachea is one of the rarest sites for a PG to proliferate.^[3]^

These tumors often pose diagnostic and therapeutic challenges due to their rarity, vascularity, and potential for local invasion.

By searching PubMed using the keywords “paraganglioma,” “chemodectoma,” “trachea,” and “tracheal” as headings, we found 12 case reports conducted between 1993 and 2022.^[1,3,4,6,7,10–16]^ These cases are presented in Table 1. Among these 12 cases, 8 (67%) were male and 4 (33%) were female. The age range of patients was 8 to 78 years, with a median age of 44 years. Reports suggest that PGs are more commonly seen in females, with a male-to-female ratio of 1:3. These tumors are most frequently observed in middle-aged populations.^[17]^ Our case was older than the estimated median age of review.

**Table 1:** Reported cases of subglottic laryngeal paragangliomas.

Author	Year	Age	Sex	Size	Site	Duration of symptoms	Treatment	Follow-up
Gallimore and Goldstraw^[7]^	1993	55	F	30 × 30 mm	Anterior wall of the trachea just proximal to the carina	1 year	Segmental tracheal resection through a right thoracotomy	1-year DF
Jones et al^[15]^	2001	41	M	Not reported	Subglottis	3 weeks	Local excision	1-year DF
Maisel et al^[10]^	2003	78	F	20 mm	1.5 cm below the true vocal cords	4 days	Open local resection	3 years DF
Khalkhali et al^[12]^	2005	25	M	10 mm in the largest diameter	Lt subglottis	18 months	Open resection	2 years DF
Metzdorff et al^[11]^	2012	40	M	Not reported	Subglottis	1 year	Open resection	2 weeks DF
Hochhegger et al^[14]^	2014	35	M	Not reported	Posterior wall of the cervical trachea	4 days	Surgical resection	Not reported
Wannaz et al^[1]^	2018	52	M	20 × 20 mm	Subglottis/membranous trachea	4 months	Membranous tracheo-carinal resection	9 months DF
Dimachkieh et al^[6]^	2018	8	M	17 × 12 × 16 mm	Right subglottis	3 months	Open resection	Not reported
Hu et al^[4]^	2021	22	M	30 × 30 × 20 mm	Right subglottis	3 months	Lateral cervical resection	3 years DF
Kamada et al^[16]^	2021 2021	67 82	F M	18 × 14 mm 19 × 15 mm	Lt subglottis The membranous portion of the posterior tracheal wall	12 months A few years	Open resection, LN dissection Open resection	6 months DF 7 months DF
Verma et al^[3]^	2022	66	F	15–10 mm	3 cm below the level of the vocal cords	6 months	Laryngeal co-ablation wand resection	3 months DF

DF = disease free.

Most tracheal PGs are found in the posterior and the posterolateral parts of the membranous trachea. Only in one of the cases, the mass situated in the anterior trachea proximal to the carina.^[7]^ On gross examination, PGs are said to be firm, rubbery, sessile (although there was 1 reported case of pedunculated tracheal PG^[3]^), vascular submucosal masses that are red or blue in hue.^[10]^ The size of the tumors varied between 10 and 30 mm in diameter. Radiologic studies help assess the tumor size and determine the vascular supply and extension of the tumor to adjacent structures. Tissue biopsies are usually avoided due to the hypervascular nature of PGs, but if they are to be conducted, the involvement of a thoracic surgeon and the availability of complex airway management techniques, including rigid bronchoscopy, are warranted.^[11]^ In our review, out of 12 cases, 6 underwent a tissue biopsy.^[4,6,7,10,12,13]^ In 2 studies,^[11,12]^ the biopsy had to be terminated because of massive hemorrhage and was repeated later with hemostasis achieved by using Nd:YAG laser and pressure. Two studies^[14,15]^ did not state whether a biopsy was conducted.

PGs usually present with symptoms depending on their anatomical location. In the trachea, they present with nonspecific symptoms such as wheezing, dyspnea, hemoptysis, and cough, which makes them easy to misdiagnose, especially with airway diseases such as asthma.^[4]^ Our patient presented with dyspnea, wheezing, hoarseness, hemoptysis, and globus sensation dating back to 2 years before diagnosis. Duration of symptoms varied widely between cases, ranging between 4 days^[14]^ and 18 months.^[12]^

Management strategies were predominantly surgical, with open resection being the most commonly employed technique (in 7 cases). Radiotherapy and chemotherapy are ineffective in treating these masses. Two patients underwent laryngoscopic local resection with excision of granulation tissue using a CO_2_ laser.^[4,12]^ Right thoracotomy was the method of choice for 2 patients.^[1,7]^ For the patient with a pedunculated PG, ablation with a laryngeal co-ablation wand was successful.^[3]^ In our case, a multidisciplinary strategy was employed that involved preoperative embolization of the arteries providing blood to reduce the chances of bleeding. Removal of the mass was achieved by using the biopsy forceps and the tip of the rigid bronchoscope to extract the tumor and clear the blocked airway. The patient recovered without any incidents, and there were no indications of injury to the recurrent laryngeal nerve.

PGs are rare tumors mostly arising from the posterior tracheal wall. They most commonly occur in the fifth and sixth decades of life. Symptoms include wheezing, cough, stridor, hemoptysis, and dyspnea. The unspecific nature of these symptoms renders the diagnosis of PGs challenging and often leads to delays in diagnosis. Diagnosis is confirmed with endoscopic, imaging, and molecular techniques such as bronchoscopy, laryngoscopy, CT scans, MRI, and tissue biopsy. However, it should be noted that given the hypervascular nature of these masses, the decision to perform a biopsy should be weighed against the risk of bleeding. Surgical excision is the mainstay of treatment. Our report adds to the limited but growing body of evidence supporting embolization-assisted surgical resection for tracheal PGs. It also emphasizes the significance of interdisciplinary collaboration, especially in anatomically complex or high-risk cases.

## Author contributions

**Data curation:** AhmadReza Amiri, Sahar Rezaei.

**Investigation:** Mohammadreza Mikaeeli.

**Supervision:** Javad Jalili, Seyed Ziyaeddin Rasi Hashemi.

**Visualization:** Mahsa Karbasi.

**Writing – original draft:** AhmadReza Amiri, Sahar Rezaei, Mohammadreza Mikaeeli.

**Writing – review & editing:** Javad Jalili, AhmadReza Amiri, Seyed Ziyaeddin Rasi Hashemi, Mahsa Karbasi, Sarah Vaseghi.
